# Heat flow saturate of Ag/MgO-water hybrid nanofluid in heated trigonal enclosure with rotate cylindrical cavity by using Galerkin finite element

**DOI:** 10.1038/s41598-022-06134-6

**Published:** 2022-02-10

**Authors:** Fares Redouane, Wasim Jamshed, S. Suriya Uma Devi, M. Prakash, Nor Ain Azeany Mohd Nasir, Zakia Hammouch, Mohamed R. Eid, Kottakkaran Sooppy Nisar, A. Belhadj Mahammed, Abdel-Haleem Abdel-Aty, I. S. Yahia, Emad M. Eed

**Affiliations:** 1 LGIDD, Ahmed ZABANA University, Relizane, Algeria; 2 Department of Mathematics, Capital University of Science and Technology (CUST), Islamabad, 44000 Pakistan; 3 Department of Mathematics, KPR Institute of Engineering and Technology, Coimbatore, 641407 India; 4 Department of Mathematics, Dr. N.G.P Institute of Technology, Coimbatore, India; 5 Department of Mathematics, Centre for Defence Foundation Studies, Universiti Pertahanan Nasional Malaysia, Kem Sungai Besi, 57000 Kuala Lumpur, Malaysia; 6 Département Des Sciences, École Normale Supérieure, Moulay Ismail University of Meknès, 50000 Meknes, Morocco; 7 Department of Mathematics, Faculty of Science, New Valley University, Al-Kharga, Al-Wadi Al-Gadid, 72511 Egypt; 8 Department of Mathematics, Faculty of Science, Northern Border University, Arar, 1321 Saudi Arabia; 9 Department of Mathematics, College of Arts and Sciences, Prince Sattam Bin Abdulaziz University, Wadi Aldawaser, 11991 Saudi Arabia; 10 Department of Physics, College of Sciences, University of Bisha, P.O. Box 344, Bisha, 61922 Saudi Arabia; 11 Physics Department, Faculty of Science, Al-Azhar University, Assiut, 71524 Egypt; 12 Laboratory of Nano-Smart Materials for Science and Technology (LNSMST), Department of Physics, Faculty of Science, King Khalid University, P.O. Box 9004, Abha 61413, Saudi Arabia; 13 Research Center for Advanced Materials Science (RCAMS), King Khalid University, P.O. Box 9004, Abha, 61413 Saudi Arabia; 14 Nanoscience Laboratory for Environmental and Biomedical Applications (NLEBA), Semiconductor Lab., Department of Physics, Faculty of Education, Ain Shams University, Roxy, Cairo, 11757 Egypt; 15 Department of Clinical Laboratory Sciences, College of Applied Medical Sciences, Taif University, P. O. Box 11099, Taif, 21944 Saudi Arabia

**Keywords:** Mathematics and computing, Physics

## Abstract

MHD Natural convection, which is one of the principal types of convective heat transfer in numerous research of heat exchangers and geothermal energy systems, as well as nanofluids and hybrid nanofluids. This work focuses on the investigation of Natural convective heat transfer evaluation inside a porous triangular cavity filled with silver-magnesium oxide/water hybrid nanofluid [H_2_O/Ag-MgO]^hnf^ under a consistent magnetic field. The laminar and incompressible nanofluid flow is taken to account while Darcy–Forchheimer model takes account of the advection inertia effect in the porous sheet. Controlled equations of the work have been approached nondimensional and resolved by Galerkin finite element technique. The numerical analyses were carried out by varying the Darcy, Hartmann, and Rayleigh numbers, porosity, and characteristics of solid volume fraction and flow fields. Further, the findings are reported in streamlines, isotherms and Nusselt numbers. For this work, the parametric impact may be categorized into two groups. One of them has an effect on the structural factors such as triangular form and scale on the physical characteristics of the important outputs such as fluidity and thermal transfer rates. The significant findings are the parameters like Rayleigh and slightly supported by Hartmann along with Darcy number, minimally assists by solid-particle size and rotating factor as clockwise assists the cooler flow at the center and anticlockwise direction assists the warmer flow. Clear raise in heat transporting rate can be obtained for increasing solid-particle size.

## Introduction

Many researchers have been investigating the characteristics of natural convection in partly heated bodies due to its significance in many engineering applications^1,2^. Chu et al.^3^ numerically tested the effect for $Pr=0.7$, $0\le Ra\le {10}^{5}$ and for the various widths and positions of the heater on localized heating in rectangular channels.Through the finite volume technique, a partlywarmed enclosure filled with nanofluid use of the finite volume technique, Oztop and Abu-Nada^4^ examined free mode convective thermal transport. Detailed experiments have been ongoing on the effects of the rays, heater height, heater position, aspect ratio of the chamber, and fractional volume of the nanoparticles on transport and fluidity. In building roof application or certain electrical heaters triangular enclosures may be used. Varol et al.^5^ adopted the numerical way of an approach based on finite difference technique over the trigonal with heated vertical wall used flow temperature as a regulator. The normal convection on the triangular cross-sectional roof was numerically explored in wintertime conditions by Asan and Namli^6^. They found a dedicated effect on temperature and flow fields of the height base ratio and radium number. Steady cases of this problem were tested for variations in Grashof numbers and height-based ratios were discussed by Akinsete and Coleman^7^ on the trigonal enclosure.The transfer of thermal and the flow of fluid will take place through an obstacle within a cavity^8–11^. The regulation of heat transfer and fluidity characteristics inside an aperture can be used with an external magnetic field in an alternative approach^12–15^.

Due to their significance in microelectronic systems, molten metal cleansers, and nuclear reactor coolers, the magnetic field effect and fluid flow have gained a lot of attention during these last year’s^16–18^. In an open chamber with a warmed spherical dented cylinder, Billah et al.^19^ examined mixed convection. Their results exposed the impact of the size of the cylinder imposed along with the ratio of heat conduction over the flowing range and thermal transport.In the study conducted by Javid et al.^20^, the effect of a magnetic field on double-diffusive convection in complex biomimetic nanoliquid propulsion in a two-dimensional divergent channel is investigated. They found that by increasing the solutalGrashof parameter, the velocity profile of nanofluid diminishes. The boundary layer flow phenomenon can be seen in the velocity profile when the magnetic and porosity factors are greater. Mebarek-Oudina et al.^21^ determined the influence of entropy and convection on magnetized hybrid nanofluid flow in a trapezoidal porous chamber having zigzagged walls. The enclosure was equipped with an adiabatic spinning cylinder located in the center. They noticed that increasing the rate of internal heat generation boosts conductive heat transfer while lowering convective heat transport. Moreover, theyobserved that The Nusselt number increases when the size of the heating element is reduced or moved to the sloped side of the cavity. Fares et al.^22^ investigated the heat transfer behavior and the free convective flow of a hydrodynamic nanofluid within a porous square enclosure. They concluded that Increasing the concentration of nanoparticles can improve the thermal conductivity of the fluid and increase the heat transfer rate. However, increasing the fluid viscosity can increase the frictional losses and weaken the convection field. Furthermore, the interaction of the nanofluid with the porous fin layers for different Ra and Da values might have varied effects on heat transfer behavior.Utilizing the finite volume method, Islam et al.^23^ fetched numerical outcomes for the warmed square chamber with included blockage by varying its size and placements across the chamber. Porous trigonal container embedded with the thin adiabatic fin were numerically exploited by Varol^24^ for parametric variations of the flow field and thermal aspects of the container.Under the magnetic field influence, titled aperture subjected to Newtonian heating were explored by Pirmohammadi and Ghassemi^25^ with the steady laminar natural convection deduced the impact of tilting angle at higher Hartmann numbers suppresses the thermal transport across over it.Hasanuzzaman et al.^26^ tried finite element discretization for testing the impact of the magnetic field on cavity chambers. Its results evident the negative impact of the magnetic field over the thermal transporting rate. In a non-Darcy permeable chamber, Fares et al.^27^ examined the magneto-free convection of a hybrid nanofluid with an inner spinning cylinder. They found that as the porous media's permeability is increased, the average Nusselt number rises significantly. The average Nusselt number rises when the Ra rises and the Hartmann number falls. As the Hartmann number grows, the rate of heat transmission slows.Mebarek-Oudina et al.^28^ studied the MHD free convection within a porous chamber, filled with MgO-Ag/Water magneto-hybrid nanofluid. The special shape of the porous cavity allowed to investigate the thermal and dynamic behavior of the flow in terms of streamlines and isotherms in the presence of an external magnetic field. The obtained results demonstrated that the fluid flow strength tends to be higher as Ra increases and drops as the magnetic field strength increases. They observed that with increasing Ra and decreasing Ha, the isotherm distributions become more concentrated.

Nanofluid, an inevitable class of fluid with exceptional thermal transferring ability by the suspended nano-level particles in the base fluid. After it enters in heat transfer field, the researcher was tempting to use its cavity works and outbreaks many crucial results, especially with the trigonal chamber geometry^29–33^. Chamber works engaged with the nanofluid incorporated with the Magnetic interactions were studied significantly in^34,35^. Hassan et al.^36^ discussed the boundary layer flow response for a non-Newtonian hybrid nanofluid over a moving wedge. Equal suspensions of MWCNTs and SiO2 nanoparticles in a binary mixture of EG–water have been used to produce the hybrid nanofluid.The results reveal that the velocity profile is lowered as the viscosity of the nanoparticle volume fraction increases. The numerical study performed by Song et al.^37^ aims to investigate the thermal features of a hybrid nanoliquid in the flow of a power-law non-Newtonian fluid constrained by a moving belt.Using silicon dioxide (SiO2) and MWCNTs in water and EG materials, the thermal assessment of hybrid nanofluid is accessed. The obtained results showed that the homogenous suspension of SiO2 and MWCNTs in water and EG base materials is very effective in improving thermal performance. Moreover, they observed that the velocity of the hybrid nanofluid decreases as the volume fraction of nanoparticles increases. Dogonchi et al.^38^ investigated the thermal and the entropy generation of buoyancy-driven magnetic Fe_3_O_4_–H_2_O nanofluid flow within a porous enclosure with two inner obstacles.For the governing equations, a Finite element technique was used, and the shape factor of multiple particle shapes has been taken into account. The obtained findings exhibited that the inclusion of porous matrix decelerates the entropy generation. Furthermore, they mentioned that the heat transfer rate improves better in the case of the Platelet shape of nanoparticles. Hassan et al.^39^ analyzed the impacts of natural convection flow of viscoelastic polydimethylsiloxane non-Newtonian nanofluid under the addition of nanoparticles of various materials passing through a vertical cone. They noticed that the temperature and heat transfer rate coefficient rise as the thermal conductivity of the base fluid increases.In addition, they indicated that particles with a smaller radius efficiently raise intensively the temperature of the liquid compared to particles with a larger radius. Moreover, the volume concentration of particles increases the viscosity of the fluid, and as a result, the fluid's velocity slows.Molana et al.^40^ analyzed the natural convection behavior of Fe_3_O_4_ water based nanofluids inside a special porous cavity. In their work, a novel MFD viscosity model (magnetic field dependent) was applied. They noticed that the blade form of nanoparticles had a higher heat transmission rate than other shapes such as platelet and spherical.

Epical results of a kind, that the physical parameters like nanoparticle fractional volume, Rayleigh coefficients, and Hartmann values can regulate the thermal transfer rates under magnetically circumstances were derived by Ghasemi et al.^41^ under free convection mode.In a trapezoidalenclosure packed with nanofluid, Mahmoudi et al.^42^ obtained the numerical solutions for natural convection MHD. The effect on thermaltransport and fluidity of the Rayleigh digit, Hartmann number, and volume fraction for nanoparticles have been studied. Eshaghi et al.^43^ accomplished a numerical simulation to examine the DDNC (Double diffusive natural convection) of a Cu–Al_2_O_3_/H_2_O hybrid nanoliquid within an H-shaped chamber with a baffle on the top wall. The obtained results demonstrated that increasing the buoyancy ratio and lowering the Lewis number increased the average Nusselt number. Belhadj et al.^44^ conducted a numerical study of MHD natural convection of a triangular cavity involving Ag–MgO/Water hybrid nanoliquid. The triangular cavity was equipped with an inner rotating obstacle, and a quarter circular porous media at its right-angled corner. They found that the existence of the magnetic effect, which is evaluated by the Hartmann number, has a regulating effect on reducing of heat transfer. For greater Ra and lower Ha, the highest rate is reached. In addition, they noticed that the increment of the nanoparticle’s concentration in the working fluid boosts heat transport within the cavity. Afshar et al.^45^ examined the nanofluid natural convection and the entropy production within a porous chamber filled with nano-encapsulated phase change materials (NEPCMs) with a volumetric heat sink. They concluded that the heat transfer improves by the combination of heat capacity and thermal conductivity for fusion temperature, as well as the increment of the solid fraction inside the working fluid. Brahimi et al.^46^ evaluated the mobility and the thermal behavior of a mixed water-based hybrid nanofluid of silver and magnesium oxide in a porous enclosure, subjected to magnetic forces. In their work, the efficient Galerkin Finite Element method (GFEM) was used to solve the governing model equations. The findings revealed that, in addition to flow-influencing restrictions, the enclosure's meandering shape of the cavity has a considerable influence on the fluidity of the working hybrid nanoliquid.

Tracked back on the above literature path, to the best of our expertise MHD was not listed in works of these kinds. Through the novel studied geometry, the aim of the present work is the evaluation of the natural waved convection of a partly heated nanofluid trigonal enclosure with an inner revolving adiabatic cylinder. The purpose of this analysis is to analyze the traces on the flow and heat transport of fluid by the Rayleigh number, Hartmann count, fractional volume of solid particles from the nano-fluid, and angular cylindrical rotation speed. Analysis of the second law is also carried out. The findings are described for the various parameter configurations employing straightening lines, isotherms, output, and local and averaged Nusselt numbers.

## Mathematical drafting

**Figure 1:**
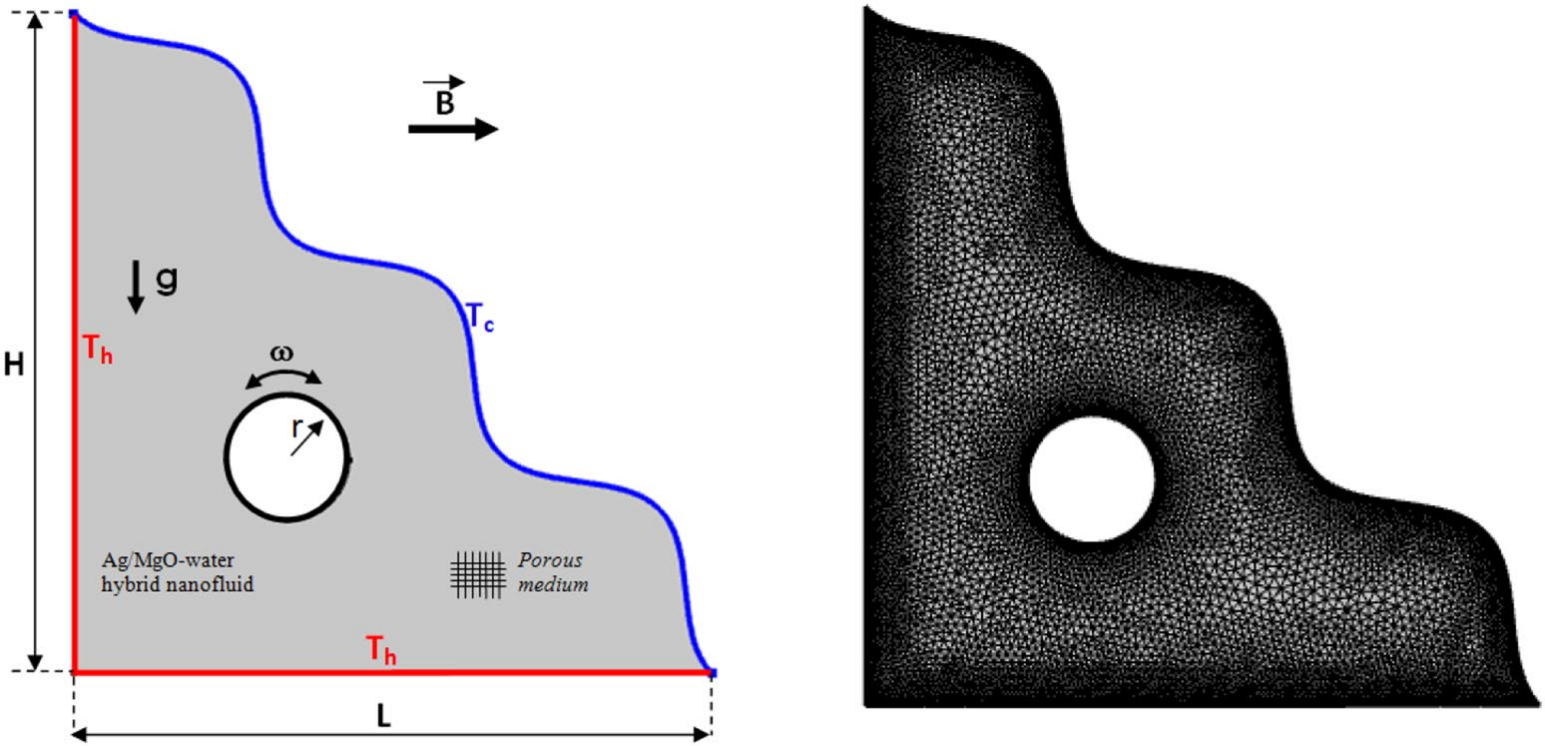
Boundary conditions and mesh of the studied model.

The representational sketch with physical aspects of the problem was portrayed in Fig. 1. Trigonal chamber with lateral and vertical walls warmed and preserved at a temperature $T_{h}$ and slanting wavy wall retains as the cooler temperature $T_{c}$. Rotating isentropic cylinder with radius $r$ and angular rotational speed $\omega$ embedded at the center of the chamber loaded with [H_2_O/Ag + MgO]^hnf^. Flow physics was framed as to be two dimensional, consistent, and stratified under influencing the lateral magnetic field. Major technical properties were considered to be consistent excludes density manipulation is considered. Thermo-physical properties of [H_2_O/Ag + MgO]^hnf^ at the reference temperature are presented in Table 1. The governed equational representation for continuity, momentum, and temperature equations adapted from^47,48^.

**Table 1:** Thermo-physical characteristics of the nanoparticles and the fluid^46^.

Property	$C_{p}$ $\left( {{\text{J}}/{\text{kg}}\;{\text{K}}} \right)$	$k$ $\left( {{\text{W}}/{\text{m}}\,{\text{k}}} \right)$	$\rho$ $\left( {{\text{kg}}/{\text{m}}^{3} } \right)$	$\beta \times 10^{ - 5}$ $\left( {{\text{K}}^{ - 1} } \right)$	$\sigma$ $\left( {\text{s/m}} \right)$	$\alpha$ $\left( {{\text{m}}^{2} /{\text{s}}} \right)$
H_2_O	4179	0.613	997.1	21	5.5 × 10^–6^	1.47 × 10^–7^
Ag	235	429	10,500	5.4	8.1 × 10^–4^	147 × 10^–3^
MgO	879	30	3970	3.36	8 × 10^–4^	95.3 × 10^–7^

Darcy–Brinkmann–Forchheimer formula, the governing equations for the porous domain are written as following^49^:

1 $$\frac{\partial u}{{\partial x}} + \frac{\partial v}{{\partial y}} = 0,$$

2 $$\frac{1}{{\varepsilon^{2} }}\left( {u\frac{\partial u}{{\partial x}} + v\frac{\partial u}{{\partial y}}} \right) = - \frac{1}{{\rho_{hnf} }}\frac{\partial P}{{\partial x}} + \frac{{\nu_{hnf} }}{\varepsilon }\left( {\frac{{\partial^{2} u}}{{\partial x^{2} }} + \frac{{\partial^{2} u}}{{\partial y^{2} }}} \right) - \nu_{hnf} \frac{u}{K} - \frac{Fc}{{\sqrt K }}u\left| u \right|$$

3 $$\frac{1}{{\varepsilon^{2} }}\left( {u\frac{\partial v}{{\partial x}} + v\frac{\partial v}{{\partial y}}} \right) = - \frac{1}{{\rho_{hnf} }}\frac{\partial P}{{\partial x}} + \frac{{\nu_{hnf} }}{\varepsilon }\left( {\frac{{\partial^{2} v}}{{\partial x^{2} }} + \frac{{\partial^{2} v}}{{\partial y^{2} }}} \right) - \nu_{hnf} \frac{v}{K} - \frac{Fc}{{\sqrt K }}v\left| u \right| \,+\, \beta_{hnf} {\text{g}}\left( {T - T_{ave} } \right) + \frac{{\sigma_{hnf} }}{{\rho_{hnf} }}B_{0}^{2} v$$

4 $$(\rho c_{p} )_{hnf} \left( {u\frac{\partial T}{{\partial x}} + v\frac{\partial T}{{\partial y}}} \right) = k_{hnf} \left( {\frac{{\partial^{2} T}}{{\partial x^{2} }} + \frac{{\partial^{2} T}}{{\partial y^{2} }}} \right)$$

where $\left| u \right| = \sqrt {u^{2} + v^{2} }$. Forchheimer coefficient $F = \frac{1.75}{{\sqrt {150} \varepsilon^{3/2} }}$ represents the operative thermal conductivity of porous media saturated with nanofluid, where $K$ is the porous medium permeability and $\varepsilon$ is its porosity, described as follow^31,50^:

5 $$K = \frac{{\varepsilon^{3} d_{m}^{2} }}{{150\left( {1 - \varepsilon } \right)^{2} }}$$

To reformulate the previous governing equations into non-dimensional ones, the following variables are used:

6 $$X = \frac{x}{L},Y = \frac{y}{L},U = \frac{uL}{{\alpha_{bf} }},V = \frac{vL}{{\alpha_{bf} }}, \theta = \frac{{T - T_{f} }}{{T_{h} - T_{f} }},P = \frac{{\left( {p + \rho_{bf} g_{y} } \right)L^{2} }}{{\rho_{bf} \alpha_{bf}^{2} }}.$$

Dimensionless numbers are given as follow:

7 $$Ra = \frac{{\beta_{bf} g\left( {T_{h} - T_{f} } \right)L^{3} }}{{\alpha_{bf} v_{bf} }},Ha = LB_{0} \sqrt {\frac{{\sigma_{bf} }}{{\mu_{bf} }}} , Da = \frac{K}{{L^{2} }},Pr = \frac{{v_{bf} }}{{\alpha_{bf} }},$$

The non-dimensional equations in the hybrid-nanofluid region can be written as^51^:

8 $$\frac{\partial U}{{\partial X}} + \frac{\partial V}{{\partial Y}} = 0,$$

9 $$\frac{1}{{\varepsilon^{2} }}\left( {U\frac{\partial U}{{\partial X}} + V\frac{\partial V}{{\partial Y}}} \right) = - \frac{\partial P}{{\partial X}} + \frac{{\rho_{f} \mu_{hnf} Pr}}{{\rho_{nf} \mu_{f} \varepsilon }}\left( {\frac{{\partial^{2} U}}{{\partial X^{2} }} + \frac{{\partial^{2} U}}{{\partial Y^{2} }}} \right) - \frac{{\rho_{f} \mu_{hnf} Pr}}{{\rho_{hnf} \mu_{f} Da}}U - \frac{{F_{C} \sqrt {U^{2} + V^{2} } }}{{\varepsilon^{\frac{3}{2}} \sqrt {Da} }},$$

10 $$\begin{aligned} & \frac{1}{{\varepsilon^{2} }}\left( {U\frac{\partial V}{{\partial X}} + V\frac{\partial V}{{\partial Y}}} \right) = - \frac{\partial P}{{\partial Y}} + \frac{{\rho_{f} \mu_{hnf} Pr}}{{\rho_{hnf} \mu_{f} \varepsilon }}\left( {\frac{{\partial^{2} V}}{{\partial X^{2} }} + \frac{{\partial^{2} V}}{{\partial Y^{2} }}} \right) \\ & \quad - \frac{{\rho_{f} \mu_{nf} Pr}}{{\rho_{nf} \mu_{f} Da}}V - \frac{{F_{C} \sqrt {U^{2} + V^{2} } }}{{\varepsilon^{\frac{3}{2}} \sqrt {Da} }}V + \frac{{(\rho \beta )_{hnf} }}{{\rho_{hnf} \beta_{f} }}Ra\Pr \theta + Ha^{2} \Pr V, \\ \end{aligned}$$

11 $$U\frac{\partial \theta }{{\partial X}} + V\frac{\partial \theta }{{\partial Y}} = \frac{{k_{hnf} }}{{k_{f} }}\frac{{(\rho C_{p} )_{f} }}{{(\rho C_{p} )_{hnf} }}\left( {\frac{{\partial^{2} \theta }}{{\partial X^{2} }} + \frac{{\partial^{2} \theta }}{{\partial Y^{2} }}} \right).$$

The thermal conductivity, heat capacity, and densityof the hybrid-nanofluid can be calculatedas following^49^:

12 $$\alpha_{hnf} = \frac{{k_{hnf} }}{{(\rho C_{p} )_{hnf} }}$$

13 $$\phi = \phi_{Ag} + \phi_{MgO}$$

14 $$\rho_{hnf} = \left( {1 - \phi } \right)\rho_{f} + \left( {\phi_{Ag} .\rho_{Ag} + \phi_{MgO} . \rho_{MgO} } \right)$$

15 $$\left( {\rho C_{p} } \right)_{hnf} = \left( {1 - \phi } \right)\left( {\rho C_{p} } \right)_{f} + \left( {\phi_{Ag} .\left( {\rho C_{p} } \right)_{Ag} + \phi_{MgO} . \left( {\rho C_{p} } \right)_{MgO} } \right)$$

16 $$\left( {\rho \beta } \right)_{hnf} = \left( {1 - \phi } \right)\left( {\rho \beta } \right)_{f} + \left( {\phi_{Ag} .\left( {\rho \beta } \right)_{Ag} + \phi_{MgO} . \left( {\rho \beta } \right)_{MgO} } \right)$$

and $\phi$ is the fractional volume of nanoparticles.

The ratio of thermal conductivity of nanofluids constricted to spherical nanoparticles is approximated by the Maxwell–Garnetss^52^ model

17 $$\frac{{k_{hnf} }}{{k_{bf} }} = \frac{{k_{np} + 2k_{bf} - 2\left( {k_{bf} - k_{np} } \right)\phi }}{{k_{np} + 2k_{bf} + \left( {k_{bf} - k_{np} } \right)\phi }}$$

The effective dynamic viscosity based on the Brinkman model^53^ is considered as

18 $$\mu_{hnf} = \frac{{\mu_{bf} }}{{\left( {1 - \left( {\phi_{Ag} + \phi_{MgO} } \right)} \right)^{2.5} }}$$

For the computational domain of proper boundary conditions, the dimensional form represented for walls as follows:

- Vertical and low walls:
  19 $$u = v = 0,\quad T = T_{h}$$
- The Inclined wavy Wall:
  20 $$u = v = 0,\quad T = T_{c}$$
- Over the cylinder surfaces:
  21 $$u = - \omega \left( {y - y_{c} } \right),\quad v = - \omega \left( {x - x_{c} } \right),\quad \frac{\partial T}{{\partial n}} = 0.$$

The relationships between the velocity and the stream function are^47^:

22 $$\left. {\begin{array}{*{20}c} {U = \frac{\partial \psi }{{\partial Y}} } \\ {V = - \frac{\partial \psi }{{\partial X}}} \\ \end{array} } \right\}$$

a single equation become,

23 $$\frac{{\partial^{2} \psi }}{{\partial X^{2} }} + \frac{{\partial^{2} \psi }}{{\partial Y^{2} }} = \frac{\partial U}{{\partial Y}} - \frac{\partial V}{{\partial X}}$$

24 $$Nu_{local} = \frac{{k_{hnf} }}{{k_{bf} }}\frac{\partial T}{{\partial y}}$$

25 $$Nu_{average} = \frac{1}{L}\mathop \int \limits_{0}^{L} Nu_{local} dL$$

## Computational methodology

**Figure 2:**
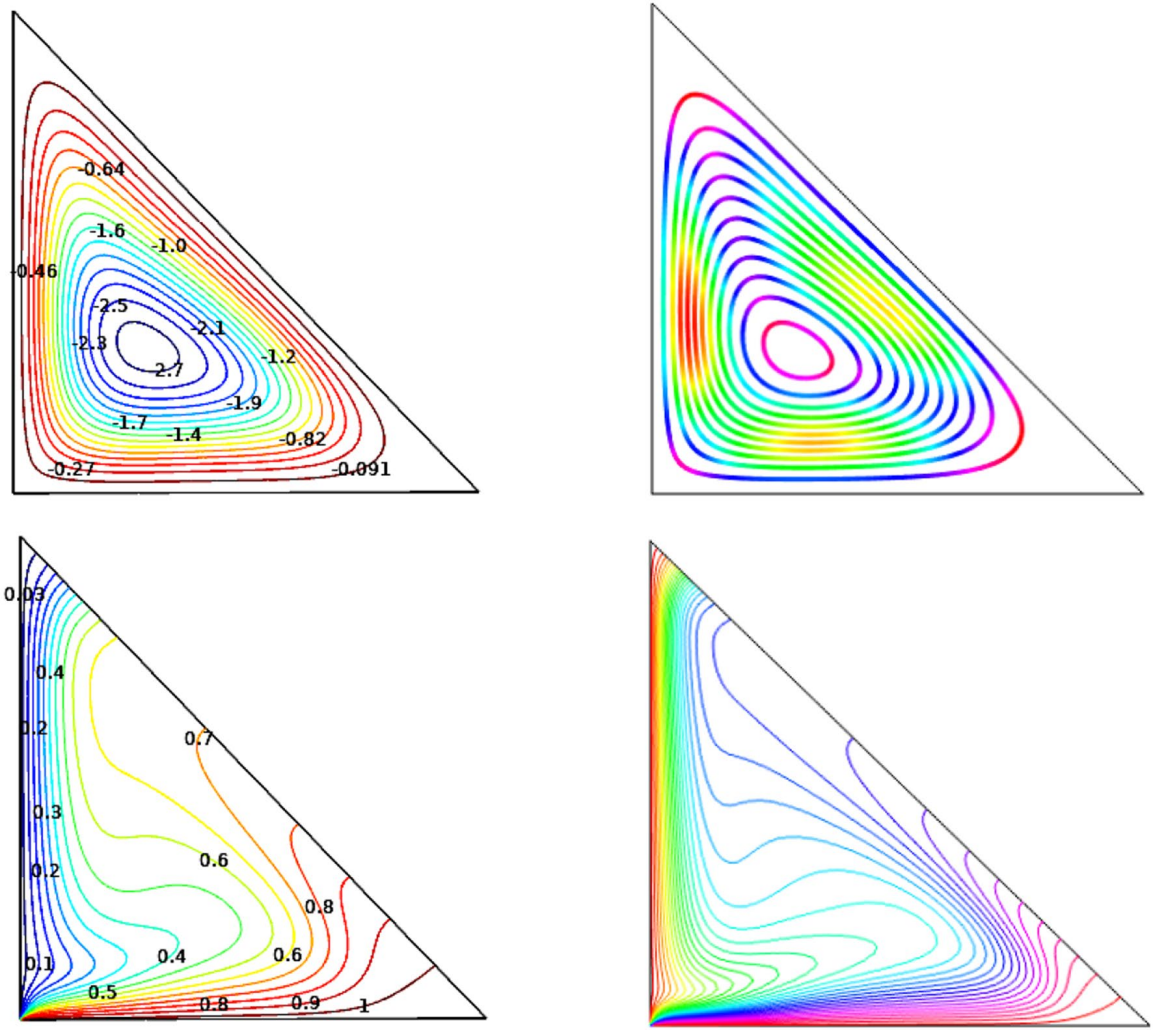
Comparison between the present work [right] and outcomes of Ref.^54^ [left] at $Ra={10}^{5}$, $Ha=0$.

An authentication test of the measurement code was performed on the obtained outcomes (Fig. 2) with those of the literature to validate the precision of the numerical results obtained in this work. Thus, the digital study of Islam et al.^54^ was considered. Six different grids were used to confirm that the results were not dependent on the grid.$Nu_{avg}$ and $\psi_{max}$ (shown in Table 2), were utilized it as the independence of flow and heat from the number of grids. Due to the variations of results, the fifth grid has opted as the terminal grid.

**Table 2:** Grid dependency of $\psi_{max}$, and $Nu_{avg}$ at the heated wall when $Ra = 10^{6}$, $Ha = 0$, $Da = 0.01$, $\varepsilon = 0.2$, $\phi = 0.02$, $r = 0.1$, $\omega = 0$ and $N = 4$.

Number of elements	1344	1622	2324	5830	16,470	19,226
$\psi_{max}$	1.2002	1.2003	1.2030	1.2065	1.2087	1.2093
$Nu_{avg}$	4.2620	4.3017	4.5410	5.4678	6.3120	6.3125

It should be remembered that the above guiding equations along with the limit conditions are determined by the system Galerkin finite element technique (GFET)^53^. The Galerkin weighted residual finite element formula solves the equations numerically. Basically, GFET starts by multiplying the weight function ω(x) and form the integral in the domain. Following that, along with the trail function, the step function is also chosen with the order of interpolation. Next, each elements were numerically evaluated by integration to get system of equations and getting the solution by solving that system.

The computer field is divided into three-way components. For any of the flow variables inside the computational domain, triangular GFET of various orders are used. Rests are obtained by substituting the approximations in the governing equations for any conservation equation. A Newton–Raphson iteration algorithm has been used to simplify the nonlinear terms in the momentum equations. When the relative error in each of the variables meets the following convergence criteria, the convergence of the solution is assumed: $\left| {\frac{{{\Gamma }^{i + 1} - {\Gamma }^{i} }}{{{\Gamma }^{i} }}} \right| \le 10^{ - 5}$, where $i$ represents the iteration number.

## Results discussion

Flow and thermal behavior of the porous triangular chamber embedded with the rotating cylinder at the center under the linear right-angled edges were heated at a temperature $T_{h}$, while the inclined edge was maintained at the constant temperature $T_{c}$ which was loaded with the [H_2_O/Ag + MgO]^hnf^ has been analyzed. The outcomes are drawn out in the form of streamlines ($\Psi$), isotherms ($T$) and Nusselt number ($Nu$) under various physical parameters. Based on the constrains like, reflecting the physical connectivity, clarity in the plot and towards the computational limitations, the parametrical values were chosen for this study. Rayleigh number ($10^{3} \le Ra \le 10^{6}$), Hartmann number ($0 \le Ha \le 100$), Darcy number ($10^{ - 5} \le Ra \le 10^{ - 2}$), nanofluid loading ($0.02 \le \phi \le 0.08$), porosity ($0.2 \le \varepsilon \le 0.8$), solid cylinder rotation ($- 4000 \le \omega \le 4000$), cylinder radius ($0.05 \le r \le 0.2$) and wavy parameter ($0 \le N \le 6$).

### Effect of Rayleigh number

**Figure 3:**
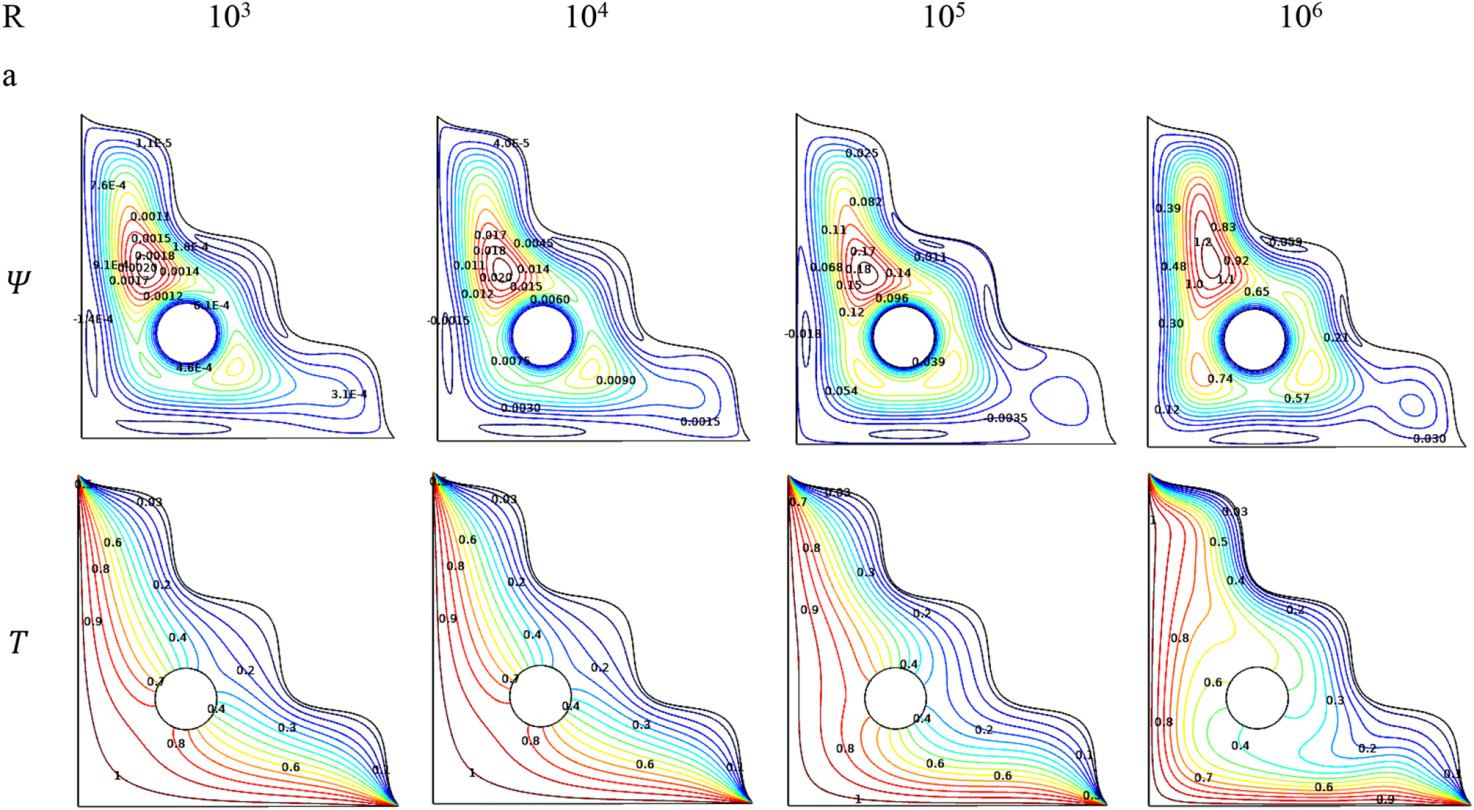
Variations of the streamlines ($\Psi$) and isotherms ($T$) with various Rayleigh number ($Ra$), $Da=0.01$, $\varphi =0.02$, $\varepsilon =0.2$,$N=4$,$r=0.1$ and $\omega =0$.

The outcomes were visualized through streamlines along with the isotherms which were used to trace the thermal variations across the chamber were shown in Fig. 3. Physically, improving Rayleigh number (Ra) leads to change in the state of flow fluid from laminar to turbulent. Initially for lower Rayleigh numbers, while the flow is being laminar, the streamlines are smoothly stressed all over the triangular chamber with smaller contours above the center cylinder. As the Rayleigh number ($Ra$) gets increased, the contour gets expanded in the first place, the smoother streamlines start to cover the vertical region of the triangular chamber and spreads around the cylinder and also a wavy lower edge of the triangular chamber.

Isotherms of the triangular chamber for the Rayleigh number ($Ra$) variations evident through the thermal distribution exerts over it. Stronger isotherms were found near the heated edge of the triangular chamber and it gets cooler as it propagates towards the wavy edge. Lower Rayleigh numbers make the flow behaves laminar which makes the thermal drive slower across the chamber this evident through the weaker isotherms tends to stay around the circular cylinder. As its values get hiked and the turbulence nature make isotherms stronger due to intense thermal transference process and move away from the cylinder and spread to nearly cover the geometry of the chamber.

**Figure 4:**
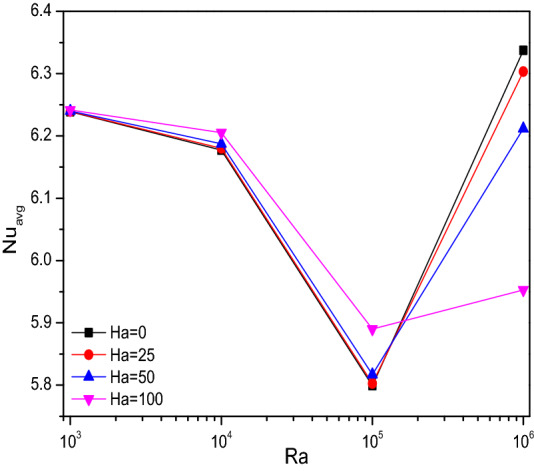
Variations of ${Nu}_{avg}$ with $Ra$ for diverse $Ha$ when $Da=0.01$, $\phi =0.02$, $\omega =0$, $N=4$, $r=0.1,$ and $\varepsilon =0.2$.

**Figure 5:**
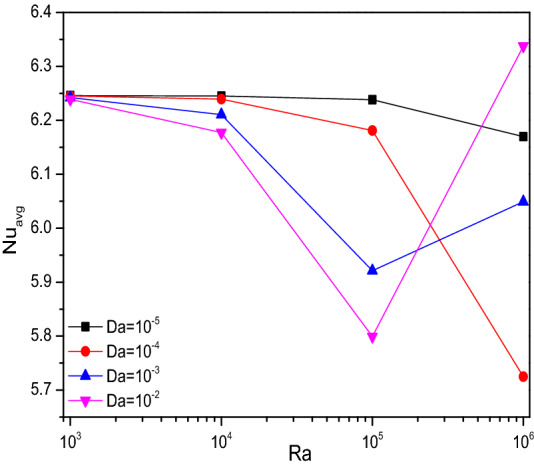
Variations of ${Nu}_{avg}$ with $Ra$ for diverse $Da$ when $Ha=0$, $\phi =0.02$, $\omega =0$, $N=4$, $r=0.1,$ and $\varepsilon =0.2$.

**Figure 6:**
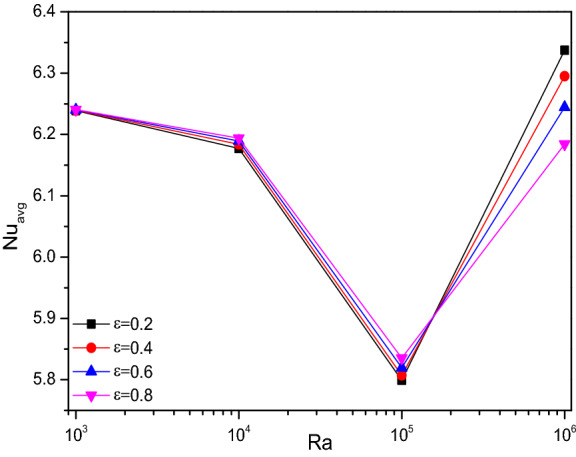
Variations of ${Nu}_{avg}$ with $Ra$ for diverse $\varepsilon$ when $Da=0.01$, $\phi =0.02$, $\omega =0$, $N=4$, $r=0.1,$ and $Ha=0$.

Figures 4, 5, and 6 render the thermal transporting rates for various values of Rayleigh number ($Ra$), Hartmann number ($Ha$), Darcy number ($Da$) and porosity ($\varepsilon$) respectively, through Nusselt number ($Nu$) tracing. In all the above-mentioned cases, the heat transfer race gets gradually reduced and get suddenly dropped around the values of $Ra = 10^{5}$ and starts fluctuating from thereafter due to the fact of turbulent flow transition for higher values of (Ra). Increasing Darcy number (Da) and Porosity ($\varepsilon$) reflects in improved permeability which allows more fluid that boosts average Nusselt number (Nu). As mentioned above these trends gets fluctuated with the turbulent behavior.

**Figure 7:**
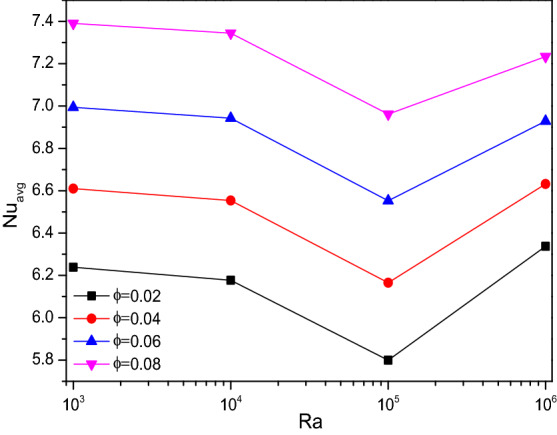
Variations of ${Nu}_{avg}$ with $Ra$ for diverse $\phi$ when $Da=0.01$, $Ha=0$, $\omega =0$, $N=4$, $r=0.1,$ and $\varepsilon =0.2$.

Interestingly, steady thermal transfer rates can be evident for the higher Raleigh numbers where drops at 10^5^ can be visualized for nanofluid loading ($\phi$) velocities in Fig. 7. As the fractional volume of [H_2_O/Ag + MgO]^hnf^ and starts its actual utility to improve the thermal transferring ability of the flow fluid towards the heat transporting rate, which also overcomes the fluctuations in the curve at higher values of Rayleigh number ($Ra$).

**Figure 8:**
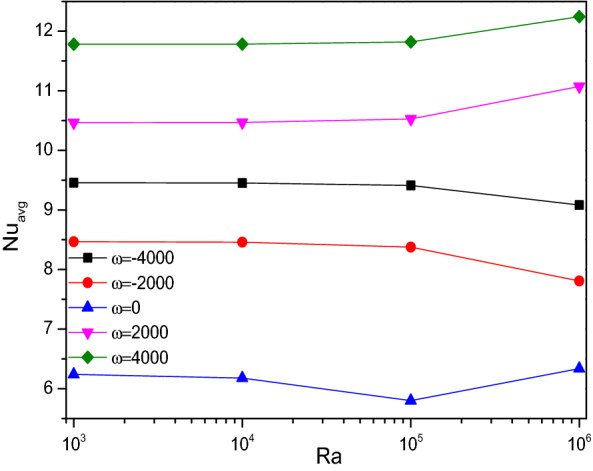
Variations of ${Nu}_{avg}$ with $Ra$ for diverse $\omega$ when $Da=0.01$, $\phi =0.02$, $Ha=0$, $N=4$, $r=0.1,$ and $\varepsilon =0.2$.

Figure 8 evoked yet another interesting behavior of thermal transfer rate across the triangular chamber. It clearly shows the influence of the cylinder placed at the center of the chamber. When the cylinder is at rest ($\omega = 0$), the heat transfer seems to be considerably low. Whereas when it starts rotating, it gets improved because of improving better fluid flow around the chamber. Interestingly, anticlockwise rotation ($\omega > 0$) exerts better heat transfer rate which brings cooler fluid towards hotter side when compared to clockwise rotation ($\omega < 0$) which brings warmer fluid form heated side, at the higher Rayleigh numbers ($Ra = 10^{5}$).

### Effect of Hartmann number

**Figure 9:**
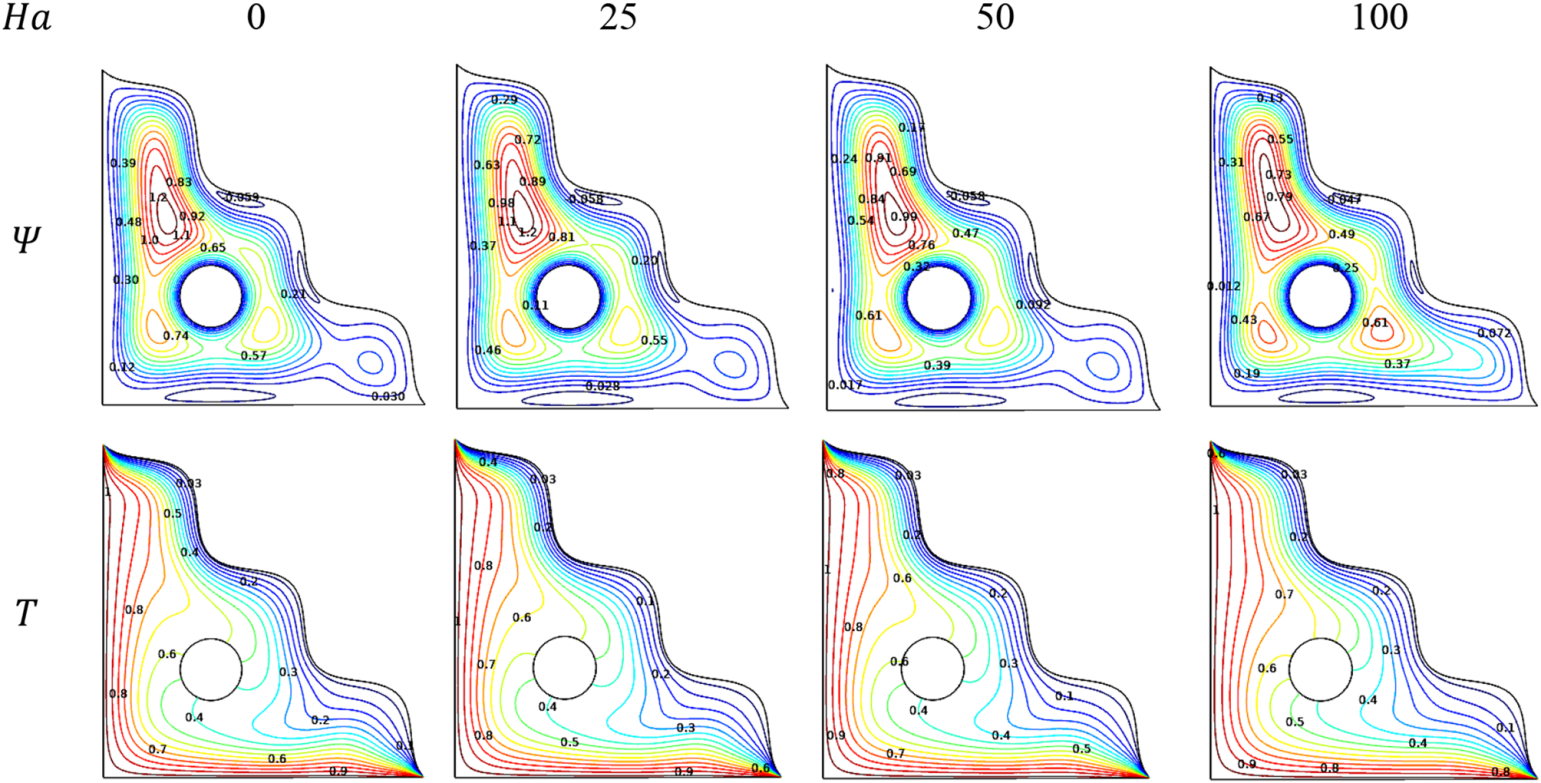
Variations of the streamlines ($\Psi$) and isotherms ($T$) with various $Ha$, $Ra = 10^{6}$, $Da = 0.01$, $\phi = 0.02$, $\omega = 0$, $N = 4$, $r = 0.1$ and $\varepsilon = 0.2$.

Across the triangular chamber, an induced magnetic field was imposed from the vertical side with linear in shape to the opposite way inclined side. Physical manipulations of the magnetic field were modeled through Hartmann number ($Ha$) variations. Such kind behavioral changes over the porous triangular chamberwere loaded with the [H_2_O/Ag + MgO]^hnf^ can be observed through Fig. 9.

In absence of a magnetic field and for its lower ranges, the streamlines formed an intense contour along with two budding contours below the cylinder. While the intense contour intensifies more for higher values of Hartmann number ($$Ha$$). The newer contours also become intense, but at a slower rate compared to the contour formed at the top of the cylinder because of the slower fluidity happened by the magnetic strength for higher Hartmann number ($$Ha)$$. It also shows the impact over the flow field by the cylinder placed at the center of the triangular chamber along with the induced magnetic influences.

**Figure 10:**
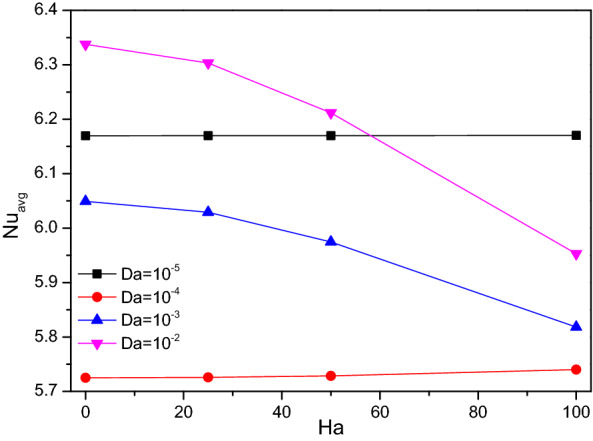
Variations of $Nu_{avg}$ with $Ha$ for diverse $Da$ when $Ra = 10^{6}$, $\omega = 0$, $\phi = 0.02$, $N = 4$, $r = 0.1$ and $\varepsilon = 0.2$.

**Figure 11:**
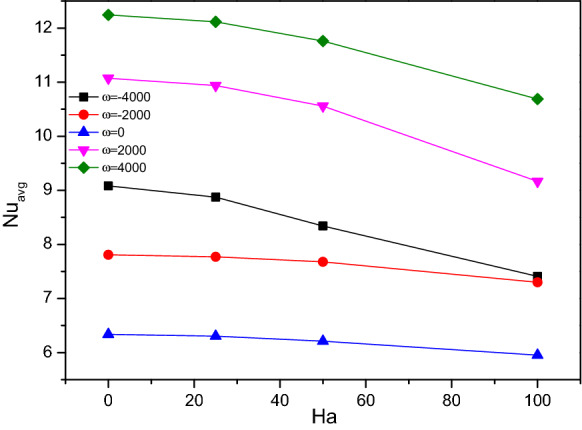
Variations of $Nu_{avg}$ with $Ha$ for diverse $\omega$ when $Ra = 10^{6}$, $Da = 0.01$, $\phi = 0.02$, $N = 4$, $r = 0.1$ and $\varepsilon = 0.2$.

Regarding isotherms, $Ha$ exerts only minimal impact over the velocity, as it is done to influence the flow transmission across the chamber linearly. Nusselt number plots for few values of $Ha$ along with the Darcy number ($Da$) were annotated through the Figs. 10 and 11. It tries to convey that; the thermal transporting rate gets reduced due to the slower fluidity for higher values of $Ha$. Especially, after $Ha = 50$ the heat transfer rate exerts fluctuations with higher $Da$ and cylindrical rotation variations.

**Figure 12:**
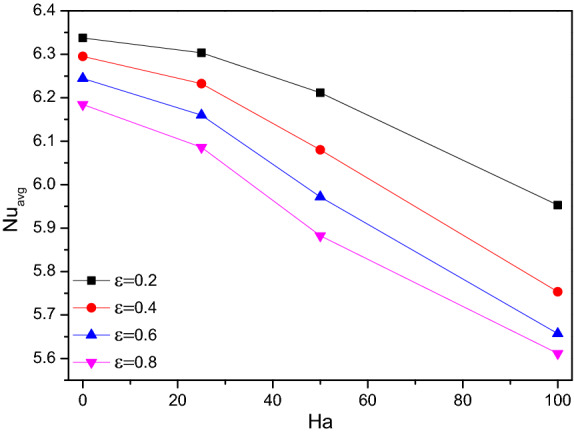
Variations of $Nu_{avg}$ with $Ha$ for diverse $\varepsilon$ when $Ra = 10^{6}$, $\omega = 0$, $\phi = 0.02$, $N = 4$, $r = 0.1$ and $Da = 0.01$.

**Figure 13:**
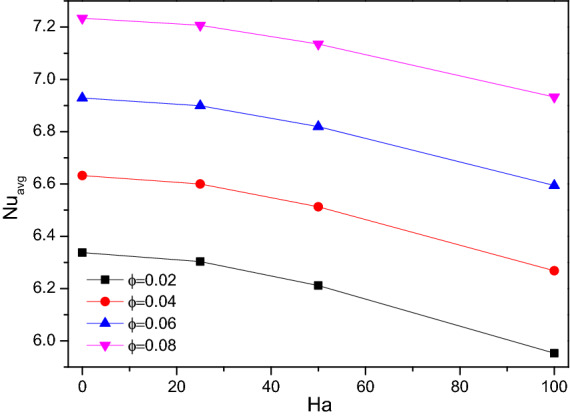
Variations of $Nu_{avg}$ with $Ha$ for diverse $\phi$ when $Ra = 10^{6}$, $\omega = 0$, $Da = 0.01$, $N = 4$, $r = 0.1$ and $\varepsilon = 0.2$.

Figures 12 and 13 illustrate the rate of Nusselt number ($Nu$), it drops for higher values of Hartmann number ($Ha$) while plotted along with the porosity ($\varepsilon$) and the hiked nanofluid loading ($\phi$) variations particularly after $Ha = 50$. While the higher Hartman number (Ha) makes the flow gentle where the increased porosity tends to drain more fluid. Both facts were against the thermal transference process. For higher values of nanofluid loading ($\phi$) reflects in improving the thermal transference ability of the flow fluid towards enhanced thermal transference rate.

### Effect of Darcy number

**Figure 14:**
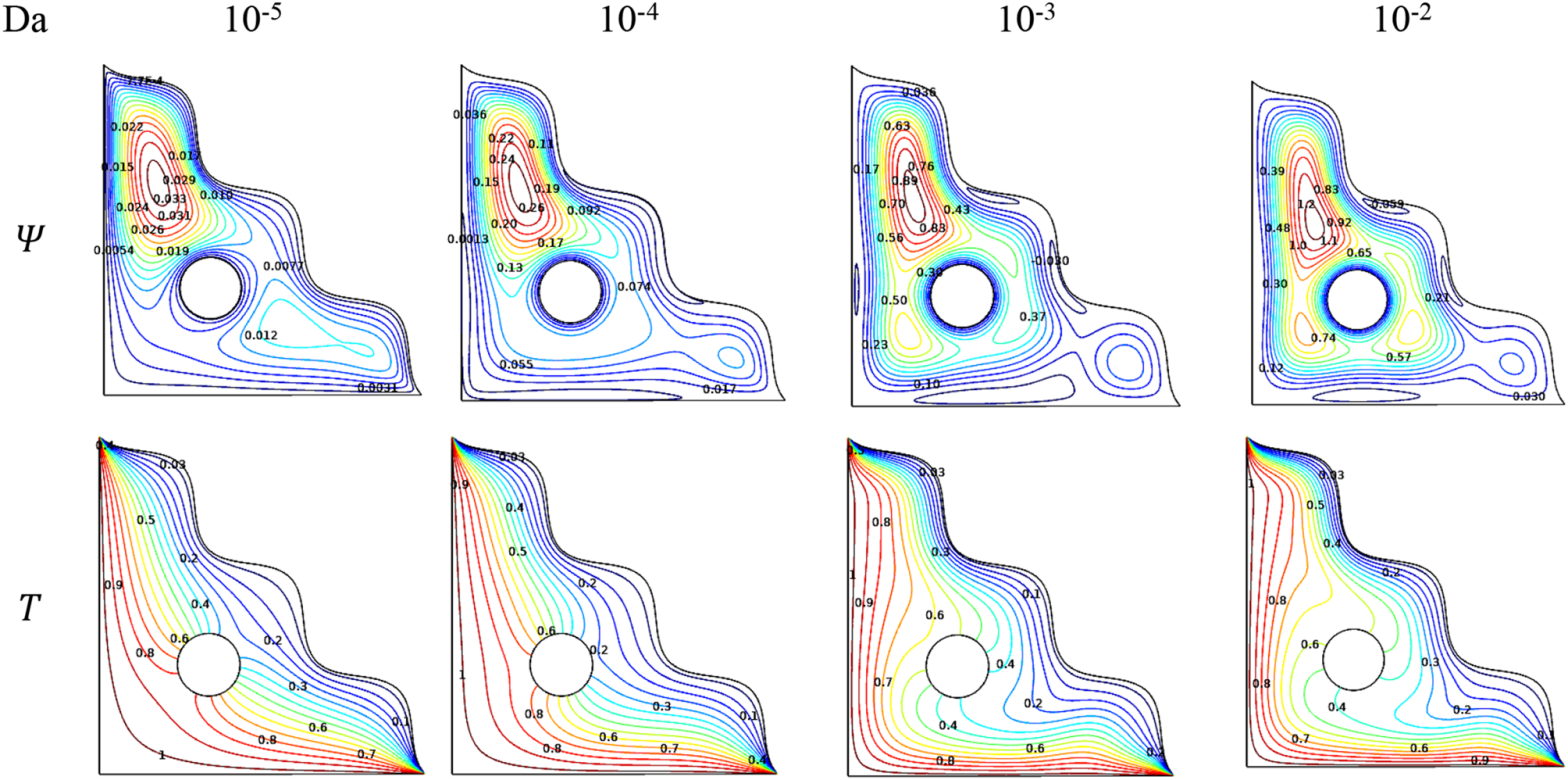
Variations of the streamlines ($\Psi$) and isotherms ($T$) with various $Ha$, $Ra = 10^{6}$, $\phi = 0.02$, $\omega = 0$, $N = 4$, $r = 0.1$ and $\varepsilon = 0.2$.

Raise in $Da$ tends to improve the permeability of the porous structure of the triangular chamber and assist the flow symmetry can be exposed through Fig. 14. As the streamlines were expanded up with one denser contour in the upper triangular region above cylinder initially at the lower $Da$ and it gets improved for intense values of $Da$, the flow changes to cover all over the chamber smoothly along with the two smaller contours.

Since the changes are engaged by the effect of thermal transport of [H_2_O/Ag + MgO]^hnf^, the permeability plays a vital role in thermal conduction of the chamber which may be due to the number of particles enters through it can manipulate the heat transfer rate all over the chamber. This claim assists to stretches near the cylinder and keeps improved to cover all over the chamber as $Da$ gets increased.

**Figure 15:**
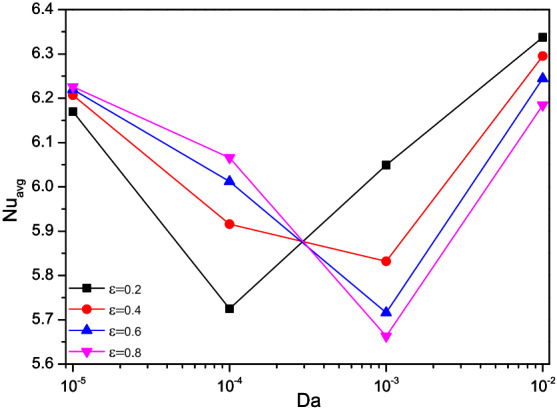
Variations of $Nu_{avg}$ with $Da$ for diverse $\varepsilon$ when $Ra = 10^{6}$, $\omega = 0$, $\phi = 0.02$, $N = 4$, $r = 0.1$ and $Ha = 0$.

**Figure 16:**
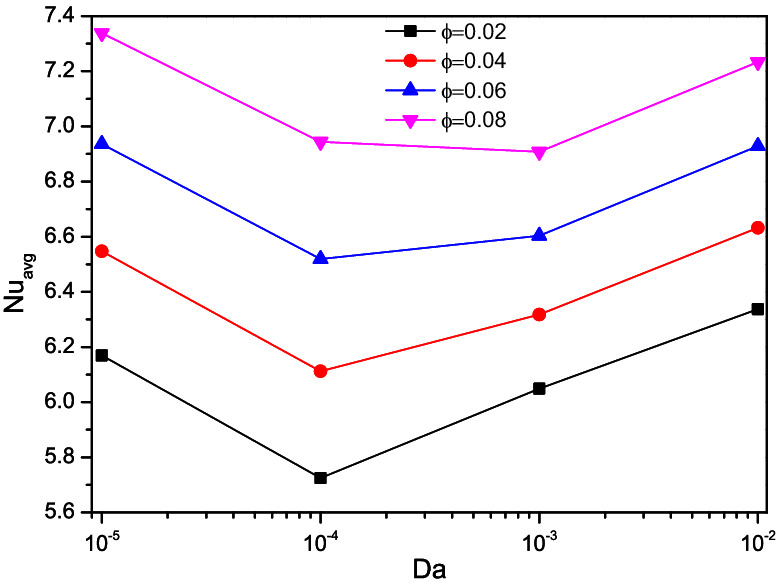
Variations of $Nu_{avg}$ with $Da$ for diverse $\phi$ when $Ra = 10^{6}$, $\omega = 0$, $\varepsilon = 0.2$, $N = 4$, $r = 0.1$ and $Ha = 0$.

**Figure 17:**
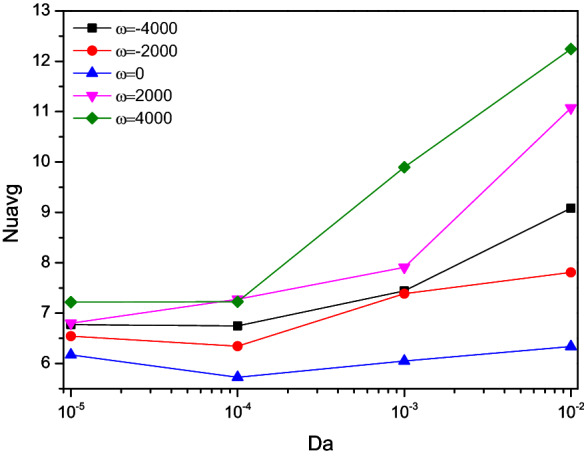
Variations of $Nu_{avg}$ with $Da$ for diverse $\omega$ when $Ra = 10^{6}$, $\varepsilon = 0.2$, $\phi = 0.02$, $N = 4$, $r = 0.1$ and $Ha = 0$.

Figures 15, 16, and 17 portray the rate of thermal transfer held over the triangular chamber concerning Darcy number variations along with the Porosity ($\varepsilon$), nanofluid loading ($\phi$), and rotational directions ($\omega )$ respectively.

The Nusselt number ($Nu$) reduces for increasing values of Darcy number ($Da = 10^{ - 4}$) and starts raised for the lower porous value ($\varepsilon = 0.2$). As the porosity gets improved, more fluid flow sets the turning point of the thermal transport rate switches to higher values of Darcy number ($Da = 10^{ - 3}$). Compare to porosity influences, nanofluid loading ($\phi$) exerts clear trends of heat transporting rate by boosting the thermal transference abilities of the flow fluid. Both of them tended to escalates the Nusselt number (Nu) for its higher values, but it has clear dents for the Darcy number ($Da = 10^{ - 4}$).

In presence of rotational variations, $Da$ promotes $Nu$ which possess some minor fluctuations between the range $10^{ - 4} \le Da \le 10^{ - 3}$. Regarding rotational direction ($\omega$), the counter-clockwise rotation ($\omega < 0$) holds the upper hand boosting cooler flow rate across the chamber heat-transmitting rate than that of clockwise direction ($\omega > 0$) and non-rotating situations ($\omega = 0$).

### Effect of nanofluid loading

**Figure 18:**
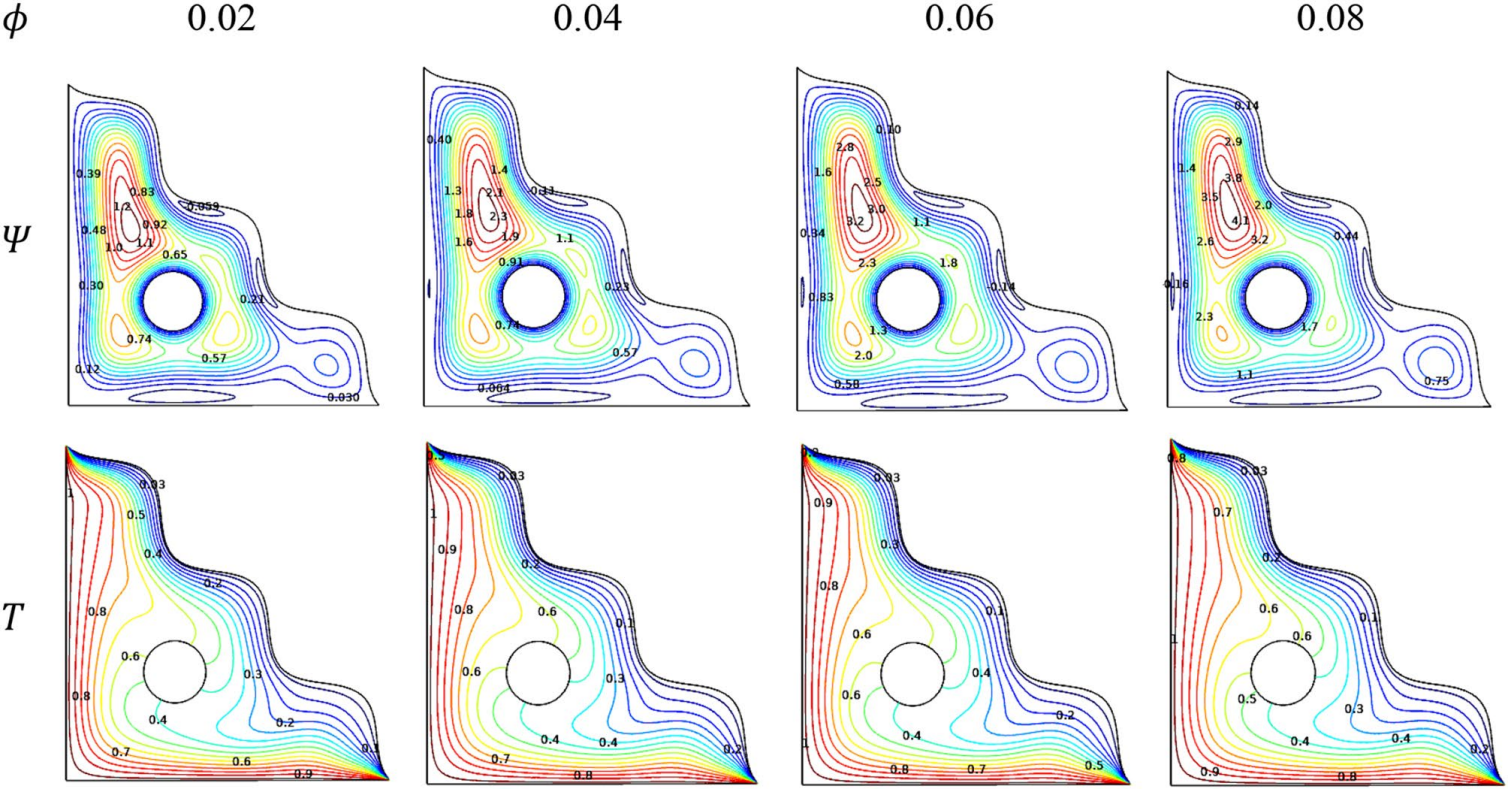
Variations of the streamlines ($\Psi$) and isotherms ($T$) with various $\phi$ at $Ra = 10^{6}$, $Ha = 0$, $Da = 0.01$, $\omega = 0$, $N = 4$, $r = 0.1$ and $\varepsilon = 0.2$.

Improving the nanofluid loading ($\phi$) facilitates the flow rates inside the chamber. It can be visualized that the contours formed around the rotating cylinder. Among them, two of those were on the weaker side for lower values of $\phi$. Later those contours turn into a strong and stretch to cover the lower chamber with better fluidity can be viewed through Fig. 18.

**Figure 19:**
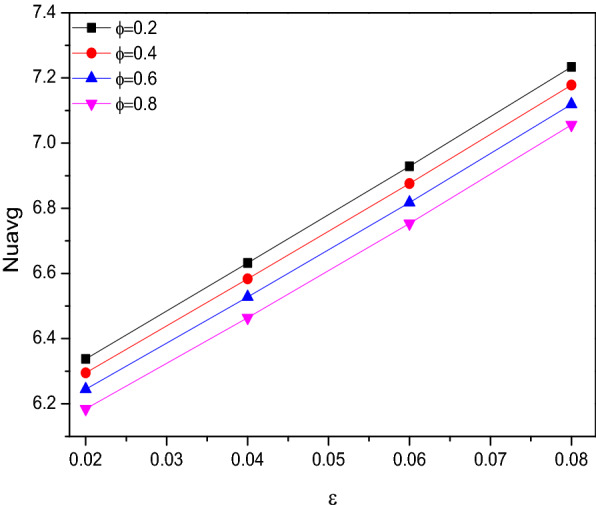
Variations of $Nu_{avg}$ with $\phi$ for diverse $\varepsilon$ when $Ra = 10^{6}$, $\omega = 0$, $Da = 0.01$, $N = 4$, $r = 0.1$ and $Ha = 0$.

Isotherms delineate the smoother heat-transmitting process due to improved loading of [H_2_O/Ag + MgO]^hnf^ inside the porous triangular chamber attracts towards the wall regions and around the circular cylinder. Figure 19 reveals the clear impact of $\phi$ on the thermal transfer rate in presence of porosity ($\varepsilon$). As the loading suppress the heat-transmitting rate, the porous values steadily escalate it to .reduces the heat transference progress.

### Porosity

**Figure 20:**
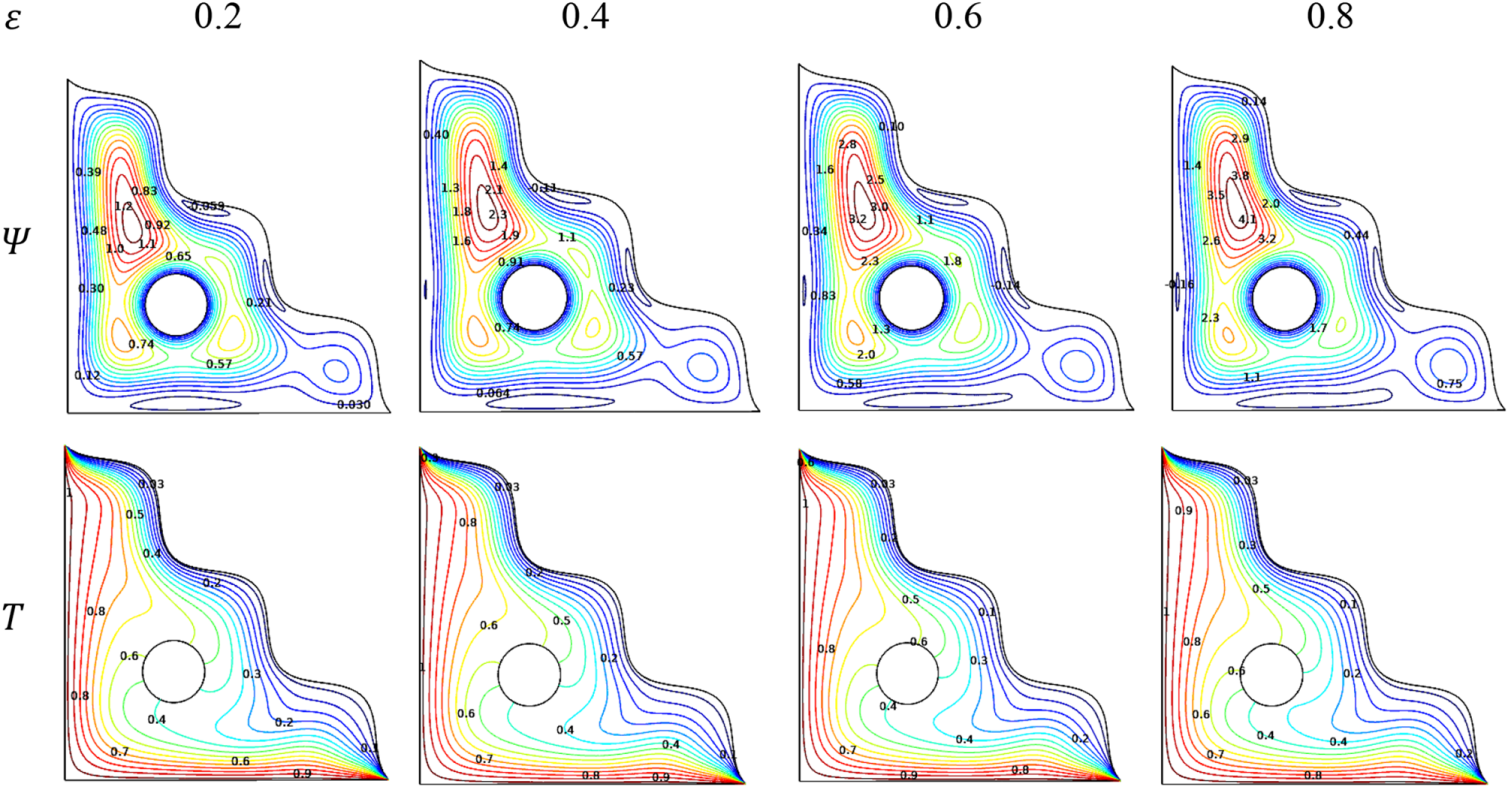
Variations of the streamlines ($\Psi$) and isotherms ($T$) with $\varepsilon$ at $Ra = 10^{6}$, $Ha = 0$, $Da = 0.01$, $\omega = 0$, $N = 4$, $r = 0.1$ and $\phi = 0.02$.

Porosity seems to be the slow motile parameter in this physical situation of the problem considered. Even for its higher values, similar contour patterns can be viewed except the minor contour variations in the lower end closer to the wavy inclined side of the triangular chamber. This may happens when the flow supposed to get through more porous structure which make it slower. Along with this, the minute variations in the isotherms for the porosity were staked as the combined plots in Fig. 20.

**Figure 21:**
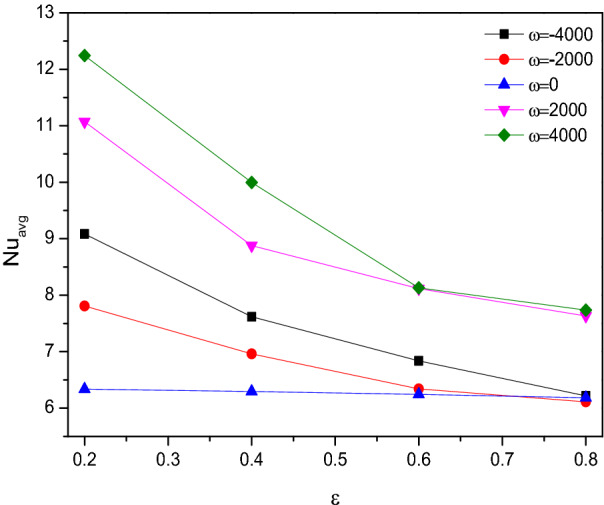
Variations of $Nu_{avg}$ with $\varepsilon$ for diverse $\omega$ when $Ra = 10^{6}$, $Da = 0.01$, $Ha = 0$, $N = 4$, $r = 0.1$ and $\phi = 0.02$.

Figure 21 looks like witness the slower impact of porosity over the Nusselt number ($Nu$) in presence of rotational direction variations both were holds the significant impact over the thermal transference across the chamber through manipulating the flow. As the embedded cylinder in the triangular chamber rotates in a clockwise direction, the thermal transfer was at the back foot until it switches its rotating direction towards the anticlockwise which exerts improved heat transfer rate.

### Effect of solid cylinder rotation

**Figure 22:**
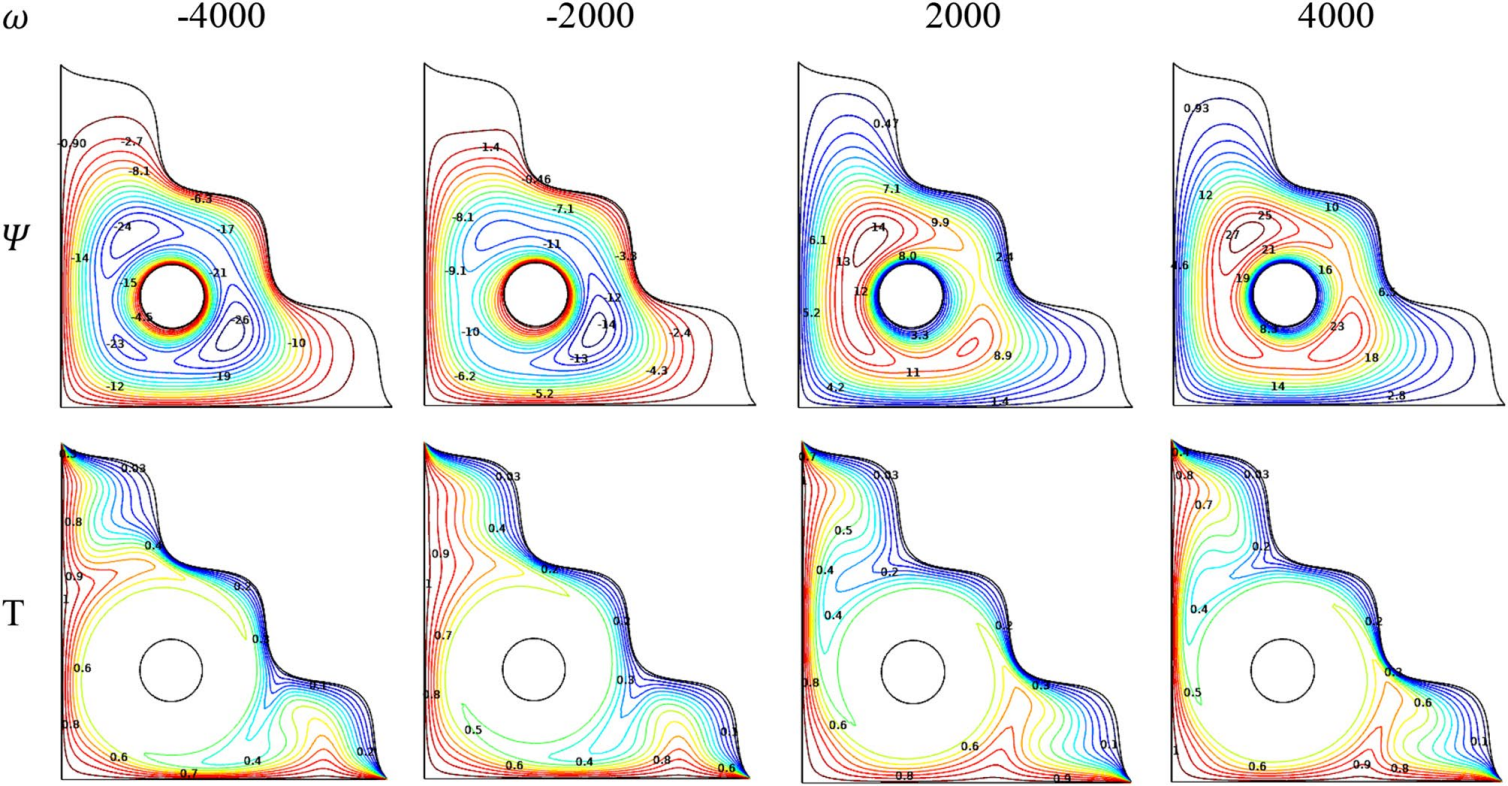
Variations of the streamlines ($\Psi$) and isotherms ($T$) with $\omega$ at $Ra = 10^{6}$, $Ha = 0$, $Da = 0.01$, $\varepsilon = 0.2$, $N = 4$, $r = 0.1$ and $\phi = 0.02$.

Streamlines (${\Psi }$) and Isotherms ($T$) for the rotation variation of the solid cylinder embedded in the center of the porous triangular chamber filled with [H_2_O/Ag + MgO]^hnf^ were divulged in Fig. 22. As expected, both the flow and thermal aspects get significant changes due to the rotation of the cylinder. As the negative rotational values ($\omega < 0$) tends to represent the clockwise rotation of the cylinder, meanwhile the counterclockwise direction ($\omega > 0$) represents through positive values. It is clear that, for clockwise rotation ($\omega < 0$) of the cylinder, the contours concentrated around the cylinder along with the faster flow fluids positioned outer and it coversthe fluidwith the lower velocity. Conversely, for the counter-clockwise rotation ($\omega > 0$), the faster fluids surrounded the cylinder which was enveloped by the slower fluids.

Regarding thermal states, the heated isotherms tend to bend towards the inner side of the chamber during the clockwise rotation, meanwhile cooler isotherms tend to move towards the center of the chamber for counterclockwise rotation.

### Effect of solid cylinder radius

**Figure 23:**
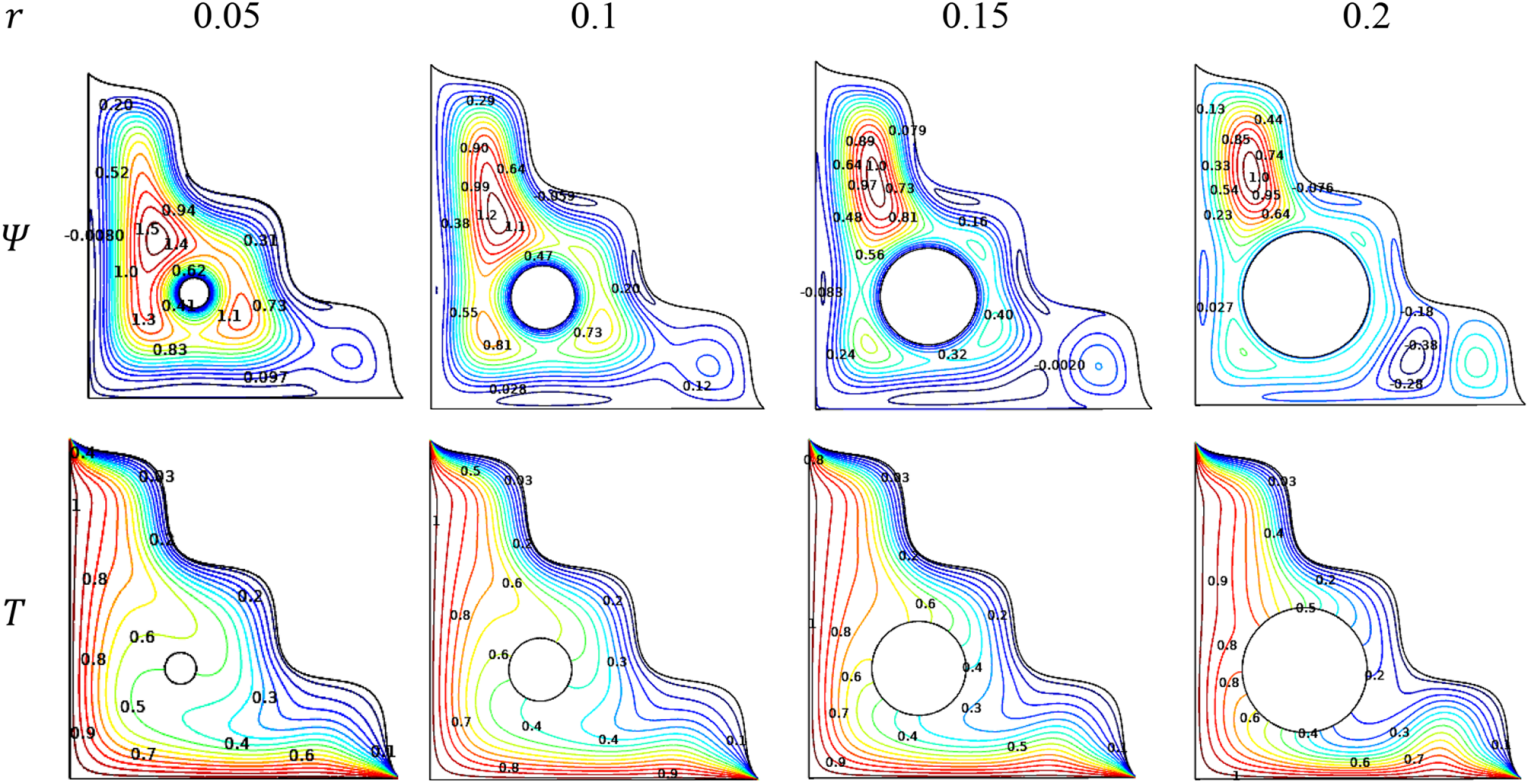
Variations of the streamlines ($\Psi$) and isotherms ($T$) with $r$ at $Ra = 10^{6}$, $Ha = 0$, $Da = 0.01$, $\varepsilon = 0.2$ and $\phi = 0.02$.

Figure 23 imparts the impression of the embedded cylinder size inside the chamber filled with [H_2_O/Ag + MgO]^hnf^. As with the rotational direction, the size of the cylinders also vital in view flow and thermal aspects. A smaller cylinder quickly rotates itself and also allows more fluid inside the chamber which creates the swift contours around it. As the cylinder enlarges in size the space for the flow motion inside the chamber gets reduced and it also takes more space in the chamber and provide lesser space to the fluid. This exhibits the weaker streamlines nearly spreads over the chamber. Because of thermal status across the triangular chamber, the smaller cylinder mixes and spreads the temperature evenly, whereas the larger cylinder helps in maintain the wall temperature such as hotter linear walls keep hotter and cooler wavy inclined wall as cooler.

**Figure 24:**
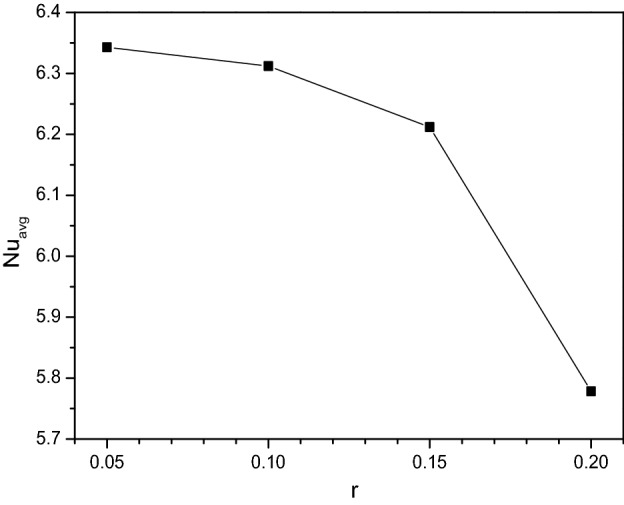
Variations of $Nu_{avg}$ with $r$ when $Ra = 10^{6}$, $Da = 0.01$, $Ha = 0$, $\omega = 0$, $N = 4$, $r = 0.1$ and $\phi = 0.02$.

In commencement with the porous discharge, the radius of the cylinder embedded in the center of the chamber slows down the thermal distribution across it. Figure 24 interprets the drop in the transferring rate of [H_2_O/Ag + MgO]^hnf^ as the cylinder expands and covers the chamber.

### Influence of wavy (N) parameter

**Figure 25:**
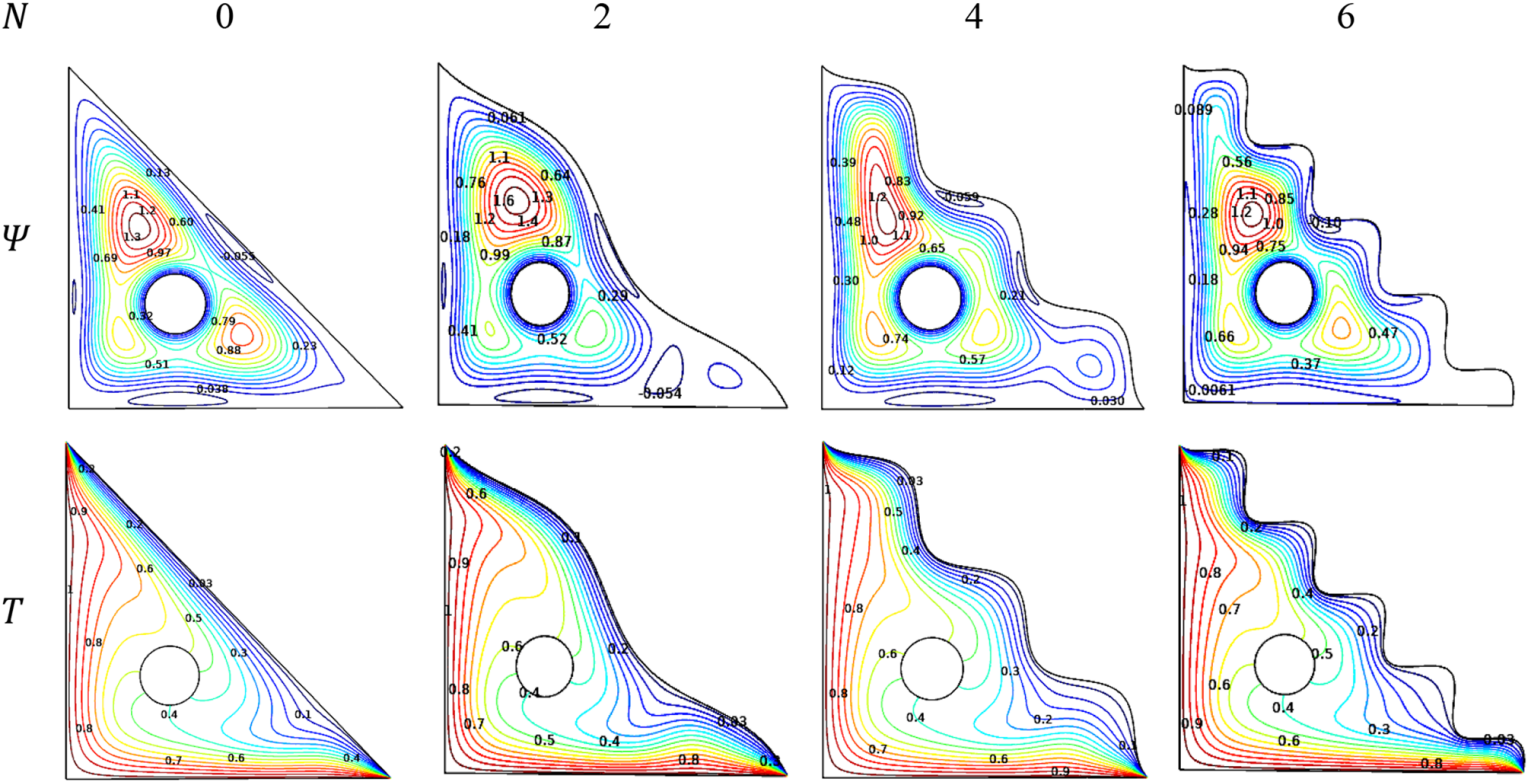
Variations of the streamlines ($\Psi$) and isotherms ($T$) with $N$ at $Ra = 10^{6}$, $Ha = 0$, $Da = 0.01$, $\varepsilon = 0.2$, $\omega = 0$ and $\phi = 0.02$.

Yet another shape influencing parameter of the triangular chamber comes into the act of flow and thermal behaviors. As minimal manipulation is done through the wavy parameter ($N$) to the inclined side of the triangular chamber. It exhibits smother fluctuations in both streamline and isotherms which are revealed through Fig. 25. But for ($N = 0$), the influence becomes interestingly higher.

Streamlines interestingly behave to the structure of the chamber in both the straight and wavy inclined sides formed the fluctuating contours for influencing values of $N$. More the wavy structure tends to have more fluctuations that make the flow slower to cover the chamber. It has a significant influence on its isotherms of the chamber as the optimal value for $N$ has to be obtained and maintained. Higher the wavy parameter tends to bring more waviness in the wall which creates more fluctuations inside the chamber which may affect its performance.

**Figure 26:**
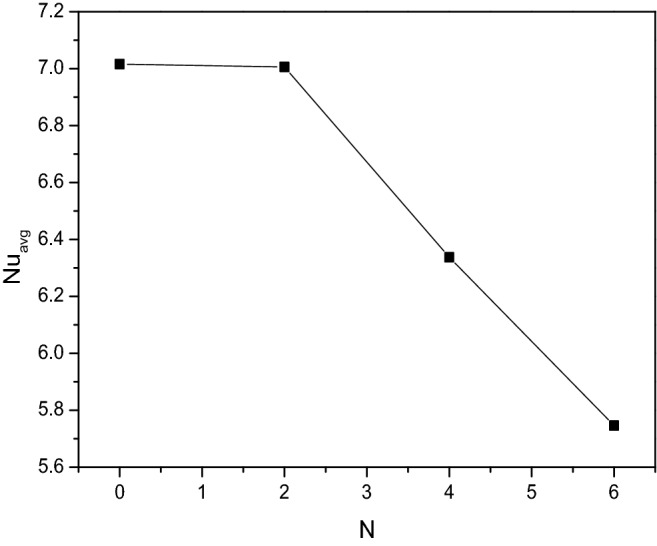
Variations of $Nu_{avg}$ with $N$ when $Ra = 10^{6}$, $Da = 0.01$, $Ha = 0$, $\omega = 0$, $r = 0.1$ and $\phi = 0.02$.

Figure 26 renders the influence of the wavy parameter ($N$) over the thermal transporting rate of the [H_2_O/Ag + MgO]^hnf^. More waviness restricts the flow and thermal motions which was the base for thermal transferring rate. It gets suppressed for the increasing values of $N$, especially after $N = 2$, the Nusselt number gets dropped suddenly because of more fluctuation in the cooler side restricts the flow from grasping heat from the other side.

## Conclusion

A parametrical study has been carried out on the porous natured triangular chamber with a wavy inclined side which has a constant cooler temperature $T_{c}$ and two linear sides heated at a temperature $T_{h}$ which is filled with the efficient [H_2_O/Ag + MgO]^hnf^ flow around the rotating cylinder embedded at the center of the chamber. Rayleigh number $Ra$, Hartmann number $Ha$, Darcy number $Da$, nanofluid loading $\phi$, porosity $\varepsilon$, solid cylinder rotation $\omega$, cylinder radius $r,$ and wavy parameter $N$ were the processing tools for the study through which the influences over the flow and thermal nature has been analyzed in terms of streamlines ${\Psi }$, isotherms $T$ and Nusselt Number $Nu_{avg}$.

The Parametric influence can be classified into two categories for this work. One is to influence the structural aspects like shape and size of the triangular chamber and on the other hand parameters influencing the physical nature of the vital outcomes of the problem like fluidity and thermal transfer rate.

Flow nature through the streamlines of [H_2_O/Ag + MgO]^hnf^ across the triangular chamber has been assisted by the parameters like $Ra$ and slightly supported by $Ha$ along with $Da$, minimally assists by $\phi$ and $\omega$ as clockwise assists the cooler flow at the center and anticlockwise direction assists the warmer flow, smaller $r$ and lower range of $N$. It gets opposed by the parameters like $\varepsilon$, larger values of $r$ and $N$.

Isotherms reflect the thermal behaviors of [H_2_O/Ag + MgO]^hnf^ in the triangular chamber with wavy edges, which was aided by the parameters like $Ra$ especially near the wavy inclined wall following that, $Ha$ express the minimal support and $Da$ along with $\phi$ which makes the thermal waves covers the chamber and lower values of cylinder radius and wavy parameter also favors the thermal movements inside the chamber. Porosity $\varepsilon$ and some higher values of the cylinder radius and wavy parameters are against it. The thermal transfer rate manipulations of [H_2_O/Ag + MgO]^hnf^ were traced in terms of $Nu$ over the triangular chamber.

Interestingly, the behavior of the thermal transport rate for parametric variations possesses fluctuations and sudden drops at some ranges of the parameters. It gets drops after the higher values of $Ra = 10^{5}$, $Ha = 50$ and after the cylinder radius with $r = 0.15$. Fluctuations in the heat transfer rates can be evident during $Da$ influence at the range of $10^{ - 4} \le Da \le 10^{ - 5}$ and higher values of $\varepsilon$ parameter. Clear raise in heat transporting rate can be obtained for increasing $\phi$ parameter.

## List of symbols

${C}_{p}$: Specific heat capacity (J kg^−1^ K^−1^)
$H, L$: Dimensionless of triangular cavity (m)
$$p$$: Pressure (Pa)
$$T$$: Temperature (K)
$U, V$: Dimensionless velocity components
$X, Y$: Dimensionless Cartesian coordinates
$$Ha$$: Hartmann number
$$Pr$$: Prandtl number
$$K$$: Permeability
$B_{0}$: Intensity of magnetic field
$$\mathrm{g}$$: Gravitational acceleration (m s^−2^)
$$k$$: Thermal conductivity (W m^−1^ K^−1^)
$$P$$: Dimensionless pressure
$$u, v$$: Velocity components (m s^−1^)
$x, y$: Dimensional Cartesian coordinates (m)
$$Ra$$: Rayleigh number
$$Nu$$: Nusselt number
$r$: Radius of cylinder
$$F_{c}$$: Forchheimer coefficient

## Greeks symbols

$$\varepsilon$$: Porosity
$$\nu$$: Momentum diffusivity
$\beta$: Thermal expansion coefficient
$$\rho$$: Density
$$\alpha$$: Thermal diffusivity
$$\phi$$: Volume fraction
$$\mu$$: Dynamic viscosity
$$\sigma$$: Electrical conductivity

## Subscripts

$$f$$: Fluid
$$np$$: Solid particles
$$loc$$: Local
$$c$$: Cold
$$hnf$$: Hybrid nanofluid
$$avg$$: Average
$$h$$: Hot
